# Accuracy of Machine Learning in Discriminating Kawasaki Disease and Other Febrile Illnesses: Systematic Review and Meta-Analysis

**DOI:** 10.2196/57641

**Published:** 2024-11-18

**Authors:** Jinpu Zhu, Fushuang Yang, Yang Wang, Zhongtian Wang, Yao Xiao, Lie Wang, Liping Sun

**Affiliations:** 1 College of Chinese Medicine Changchun University of Chinese Medicine Changchun China; 2 Center of Children's Clinic The Affiliated Hospital to Changchun University of Chinese Medicine Changchun China; 3 Beijing Jishuitan Hospital Capital Medical University Beijing China

**Keywords:** machine learning, artificial intelligence, Kawasaki disease, febrile illness, coronary artery lesions, systematic review, meta-analysis

## Abstract

**Background:**

Kawasaki disease (KD) is an acute pediatric vasculitis that can lead to coronary artery aneurysms and severe cardiovascular complications, often presenting with obvious fever in the early stages. In current clinical practice, distinguishing KD from other febrile illnesses remains a significant challenge. In recent years, some researchers have explored the potential of machine learning (ML) methods for the differential diagnosis of KD versus other febrile illnesses, as well as for predicting coronary artery lesions (CALs) in people with KD. However, there is still a lack of systematic evidence to validate their effectiveness. Therefore, we have conducted the first systematic review and meta-analysis to evaluate the accuracy of ML in differentiating KD from other febrile illnesses and in predicting CALs in people with KD, so as to provide evidence-based support for the application of ML in the diagnosis and treatment of KD.

**Objective:**

This study aimed to summarize the accuracy of ML in differentiating KD from other febrile illnesses and predicting CALs in people with KD.

**Methods:**

PubMed, Cochrane Library, Embase, and Web of Science were systematically searched until September 26, 2023. The risk of bias in the included original studies was appraised using the Prediction Model Risk of Bias Assessment Tool (PROBAST). Stata (version 15.0; StataCorp) was used for the statistical analysis.

**Results:**

A total of 29 studies were incorporated. Of them, 20 used ML to differentiate KD from other febrile illnesses. These studies involved a total of 103,882 participants, including 12,541 people with KD. In the validation set, the pooled concordance index, sensitivity, and specificity were 0.898 (95% CI 0.874-0.922), 0.91 (95% CI 0.83-0.95), and 0.86 (95% CI 0.80-0.90), respectively. Meanwhile, 9 studies used ML for early prediction of the risk of CALs in children with KD. These studies involved a total of 6503 people with KD, of whom 986 had CALs. The pooled concordance index in the validation set was 0.787 (95% CI 0.738-0.835).

**Conclusions:**

The diagnostic and predictive factors used in the studies we included were primarily derived from common clinical data. The ML models constructed based on these clinical data demonstrated promising effectiveness in differentiating KD from other febrile illnesses and in predicting coronary artery lesions. Therefore, in future research, we can explore the use of ML methods to identify more efficient predictors and develop tools that can be applied on a broader scale for the differentiation of KD and the prediction of CALs.

## Introduction

### Background

Kawasaki disease (KD) is a systemic vasculitis of small and medium-sized vessels, which has nonspecific symptoms and predominantly afflicts children aged 5 years or younger [1]. The incidence of KD worldwide varies greatly among different regions and ethnic groups, ranging from 46.3 to 359 cases per 100,000 people in East Asia, compared with 4.5 to 20 cases per 100,000 children aged 5 years or younger in Europe and North America [2]. KD is regarded as the most frequent cause of pediatric-acquired heart disease in developed countries. Its primary sequelae are related to the coronary system and may have a huge impact on the myocardium, valves, and arterial walls in some pediatric patients, which is less commonly affected in other febrile illnesses [3]. Early recognition of KD and timely administration of gammaglobulin significantly reduces the risk of coronary artery lesions (CALs), promotes the regression of coronary aneurysms, and decreases the likelihood of long-term health complications in affected children with KD [3-5].

Currently, the etiology and exact causative agent of KD remain unknown. Its clinical diagnosis is made mainly on the basis of the patient’s clinical manifestations, including fever, skin and mucosal changes, swollen lymph nodes, extremity changes, rash, and cardiac manifestations, such as coronary artery dilatation or aneurysm [3,6]. Early and accurate recognition of KD is challenging due to several factors, its clinical symptoms do not appear in a specific sequence and may not manifest simultaneously; the characteristic symptoms and laboratory findings often overlap with those of other febrile illnesses, such as respiratory infections, sepsis, and multisystem inflammatory syndrome; and its self-limited nature in the acute phase can cause symptoms to diminish or disappear before a definitive diagnosis is made [3,7,8]. People with KD who do not respond adequately to initial intravenous immunoglobulin (IVIG) therapy are at a higher risk of developing CALs. Therefore, classical scoring systems for predicting IVIG nonresponsiveness, such as the Kobayashi score [9], Harada score [10], and Egami score [11], are also considered valuable for assessing the risk of CALs. However, the sensitivity of these established scoring systems outside Japan is not good. In addition, since infectious agents and immune regulatory dysfunction in the body may be the causative factors of KD, stress-related inflammatory markers occurring after exogenous infections, such as the erythrocyte sedimentation rate (ESR), C-reactive protein (CRP) level, white blood cell (WBC) count, and platelet (PLT) count, are thought to have the ability to predict CALs in people with KD, and cardiac markers including N-terminal pro-B-type natriuretic peptide and markers of cardiomyocyte damage are also considered useful for predicting CALs. However, the predictive performance of single predictors is limited [12-14]. Therefore, at present, how to efficiently diagnose KD and how to early predict CALs in people with KD remain urgent.

Since Alan Turing proposed the theoretical foundation of artificial intelligence (AI) in the 1930s, the field has undergone over 70 years of development. Following the turn of the millennium, with the proliferation of computer technology and the internet, the surge in data availability and enhanced computational power have led to the widespread adoption of machine learning (ML) techniques [15]. Alongside advancements in medical testing technologies, vast amounts of medical imaging, laboratory monitoring data, and genetic sequencing results now require interpretation by physicians. The heavy load of medical reports and the complex communication processes between doctors and patients have contributed to diagnostic errors, inefficiencies in medical practice, and physician burnout. As a result, there is a growing clinical need for automated, intelligent, and reasonably accurate ML tools to assist in these tasks [16-18]. Classic ML techniques are broadly categorized into supervised and unsupervised learning. Compared with the latter, the former is more frequently used in the medical field because it can generate clinically applicable conclusions by learning from and analyzing large volumes of labeled data (such as laboratory data, medical images, and genetic data). This makes supervised learning particularly valuable for tasks such as disease prediction, diagnosis, and image segmentation in clinical practice [19]. ML can analyze complex data from a wide range of sources, such as clinical manifestations, laboratory results and imaging data. Unlike traditional approaches that focus on single biochemical indicators, ML can detect subtle correlations within large datasets, helping to address challenges that traditional statistical methods cannot solve. This capability enhances both predictive and diagnostic accuracy [20]. In the field of disease prediction, ML can forecast disease progression and adverse outcomes, which is crucial for developing targeted treatment and rehabilitation regimens. While, in disease diagnosis, ML is primarily applied to the diagnosis of complex diseases and the assessment of disease states. This can significantly aid in formulating specialized diagnostic strategies, thereby reducing the time and economic costs associated with diagnosis [21-24].

### Objective

In recent years, some researchers have attempted to apply ML to differentiate KD from other febrile illnesses and to predict CALs in people with KD. However, there is still a lack of systematic evidence supporting the effectiveness of ML methods in these areas, which presents challenges for the development or refinement of AI diagnostic tools. Therefore, we have conducted the first systematic review and meta-analysis to evaluate the accuracy of ML in differentiating KD from other febrile illnesses and in predicting CALs in people with KD, so as to provide evidence-based support for the future development and application of AI in these fields.

## Methods

### Study Registration

This study was executed according to the operating guidelines for systematic reviews and meta-analyses and prospectively registered on PROSPERO (CRD42023481662).

### Eligibility Criteria

The inclusion and exclusion criteria are shown in Textbox 1.

**Inclusion and exclusion criteria.**

**Inclusion criteria**

- To differentiate Kawasaki disease (KD) from other febrile illnesses, people with KD and people with febrile illnesses suspected to be KD were included as study participants. To predict coronary artery lesions (CALs) in KD, people with confirmed KD were included as study subjects.
- The types of studies included were case-control studies, cohort studies, nested case-control studies, and case-cohort studies.
- A predictive model for identifying KD or predicting CALs in people with KD was constructed entirely.
- Some studies lacked an independent validation set and only adopted k-fold cross-validation or Bootstrap. Their contribution cannot be denied. Therefore, they were also included in our systematic review.
- A small number of studies may be based on distinct machine learning (ML) models published on the same dataset, but they were also incorporated into our systematic review.
- Included studies are published in English.

**Exclusion criteria**

- Meta-analyses, reviews, guidelines, expert opinions, and similar types of studies.
- Only risk factor analysis was executed, and a full ML model was not constructed.
- Studies that did not include any of the following outcome measures for assessing the predictive accuracy of ML models: Receiver Operating Characteristic, Concordance statistic, Concordance index, sensitivity, specificity, accuracy, recovery, precision, confusion matrix, fourfold table for estimating the quality of a diagnostic test, *F*_1_-score, and calibration curve.
- Studies on the validation of mature scale.
- Studies on the predictive accuracy of single-factor predictors.

### Data Sources and Search Strategy

PubMed, Cochrane Library, Embase, and Web of Science were systematically searched until September 26, 2023. The search terms were designed based on a combination of subject headings and free-text words, without geographical restriction or time limit (year). The search strategy is detailed in Table S1 in Multimedia Appendix 1.

### Study Selection and Data Extraction

The retrieved records were imported into Endnote, and duplicate publications were excluded. Then we read the titles and abstracts to screen out the original studies that did not meet the requirements. Next, full texts were downloaded and read to select eligible studies.

A standard electronic data extraction spreadsheet was created before data extraction. The following data were extracted: titles of the original studies, the name of the first author, the year of publication, the author’s nationality, the study type, the data source, the number of people with KD, the total number of study participants, the numbers and total number of people with KD in the training set and validation set when a model was trained to identify KD, the numbers and total number of patients developing CALs in the training set and validation set when a model was trained for early prediction of CALs in KD, model validation methods, methods for handling missing values, methods for variable screening, types of models used, and predictors used in the final model.

Literature screening and data extraction were carried out independently by 2 researchers (JZ and FY), and then, the results were cross-checked. Discrepancies were resolved by the third researcher (YW).

### Risk of Bias in Studies

The risk of bias in eligible studies was appraised using the PROBAST (Prediction model Risk Of Bias Assessment Tool), which consisted of numerous questions across 4 different domains: participants, predictors, outcome, and analysis. This approach provides a comprehensive evaluation of the overall risk of bias and overall usability [25]. The 4 domains covered 2, 3, 6, and 9 specific questions, respectively. There are 3 answers (yes [Y]/probably yes [PY], no [N]/probably no [PN], and no information [NI]) to each question. If at least 1 question in a domain was answered as N or PN, it was rated at high risk. To be considered at low risk, all questions in the domain should be answered as Y or PY. The overall risk of bias was graded as low when all domains were considered at low risk, and the overall risk of bias was rated high risk when at least one domain was rated at high risk.

Risk of bias assessment was conducted independently by 2 researchers [JZ and ZW] based on the PROBAST, and then, their results were cross-checked. Discrepancies were resolved by the third researcher [LS].

### Outcome Measures

The primary outcome measures were the concordance index (C-index), sensitivity, and specificity reflecting the predictive accuracy of ML models. The secondary outcome measure was the frequency of use of a predictor to construct a ML model.

### Meta-Analysis Methods

The meta-analysis was performed using Stata (version 15.0; Stata Corp). A meta-analysis was conducted of the measure (C-index) to assess the overall accuracy of ML models. If 95% CIs and standard errors of the C-index were missing in some original studies, its standard errors were estimated with reference to a study by Debray et al [26]. A random-effects model was leveraged for meta-analysis of the C-index given the differences in variables and inconsistent parameters included in various ML models.

In addition, a bivariate mixed-effects model was leveraged for meta-analysis of sensitivity and specificity, which required that meta-analysis of sensitivity and specificity should be based on the fourfold table (2 × 2 table) for estimating the quality of a diagnostic test. However, the fourfold table was not reported in some original studies. Therefore, the fourfold table was calculated by the following 2 methods, calculation of sensitivity, specificity, and precision combined with the number of patients; and calculation of the best sensitivity and specificity determined based on the Youden index and then combined with the number of patients. When conducting a meta-analysis of the C-index, we used the “metan” program package, whereas for the meta-analysis of the fourfold table (2 × 2 table), we used the “midas” program package.

## Results

### Study Selection

**Figure 1:**
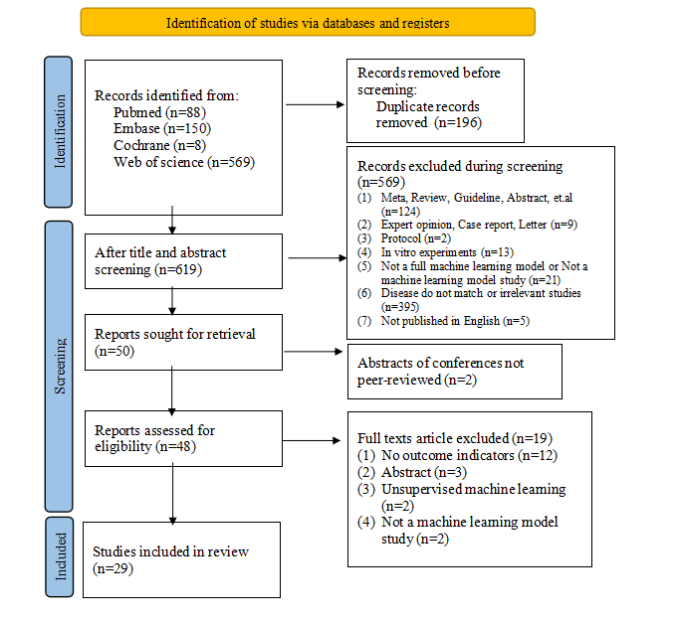
The PRISMA (Preferred Reporting Items for Systematic Reviews and Meta-Analysis) flow diagram for study selection.

A total of 815 records were searched from the databases. Of them, 196 were removed due to duplication, and 569 were excluded after screening of titles and abstracts. The full texts of the remaining 50 articles were searched and read. Of them, 2 were excluded without peer review of abstracts, and 19 were excluded for the reasons described in Figure 1. In the end, a total of 29 studies were included [27-55].

### Study Characteristics

This study included a total of 29 publications between 2001 and 2023, involving 29 ML models with variables all derived from relatively common clinical characteristics.

Twenty studies involved a total of 20 models. They, published from 2013 to 2023, focused on the differential diagnosis of KD from Febrile controls (including respiratory infections, gastrointestinal infections, urinary infections, encephalitis, multisystem inflammatory syndrome, sepsis and cervical lymphadenopathy with fever due to bacterial infections, viral infections, or indeterminate pathogens). Of them, 11 were conducted in China [27-37], 5 in the United States [38-42], 1 in Egypt [43], 1 in Germany [44], 1 in Italy [45], and 1 in South Korea [46]. These studies involved a total of 103,882 study participants, including 12,541 people with KD.

The types of models used in these 20 studies included logistic regression (9/20, 45%), support vector machine (3/20, 15%), least absolute shrinkage and selection operator (2/20, 10%), artificial neural network (2/20, 10%), extreme gradient boosting (1/20, 5%), decision tree (1/20, 5%), random forest (1/20, 5%), and linear discriminant analysis (1/20, 5%).

The other 9 studies (involving 9 models) focused on the prediction of the risk of CALs in KD children. Of them, 5 were conducted in China [47-51], 2 in Japan [52,53], 1 in Spain [54], and 1 in the United States [55]. The types of models used in these 9 studies included multivariate logistic regression (8/9, 88.89%) and an artificial neural network (1/9, 11.11%). These 9 studies involved a total of 6503 people with KD, including 986 ones with CALs. Characteristics of including studies are presented in Table S2 and Table S3 in Multimedia Appendix 1.

### Risk of Bias in Studies

**Figure 2:**
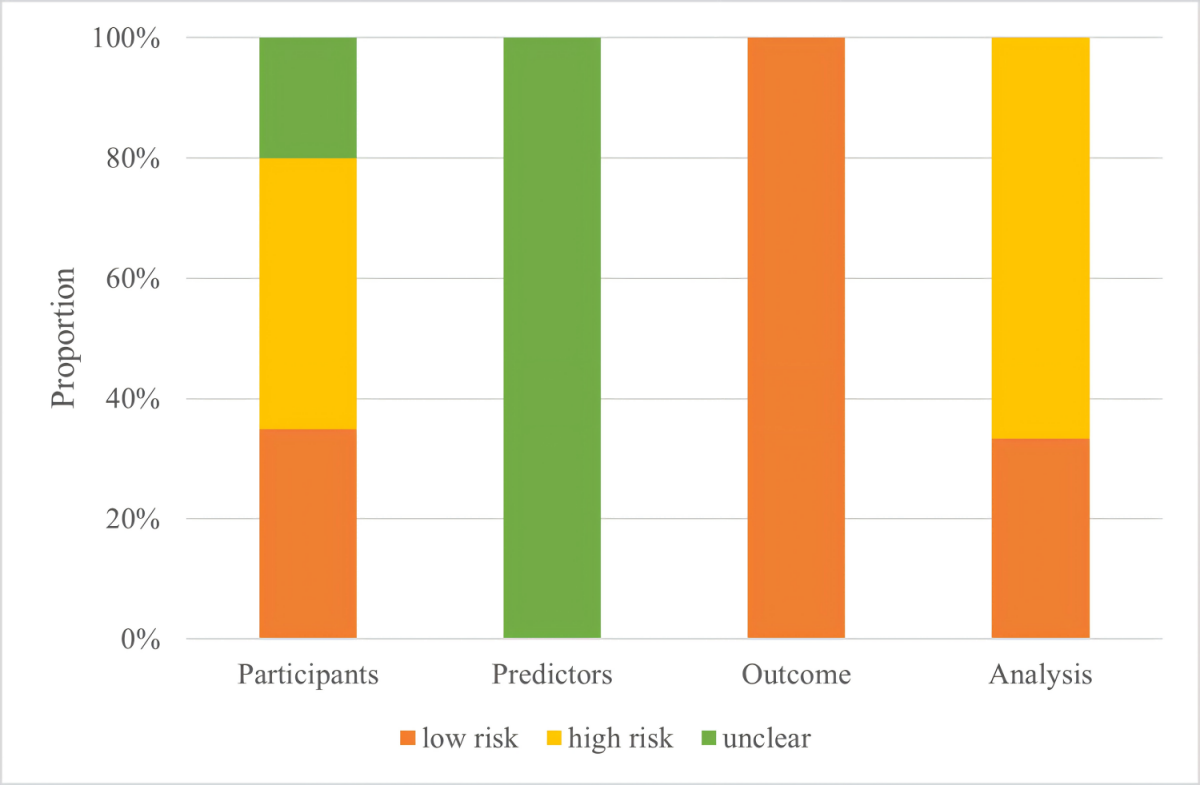
Risk assessment of machine learning models to identify Kawasaki disease from febrile controls.

In the differential diagnosis of KD from other febrile illnesses, 9 models [27-30,32,33,35,36,46] were based on data from retrospective studies, and therefore had a high risk of participant selection bias; 4 models [31,37,40,45] were constructed without data sources reported, so they were at unclear risk of bias. In the domain of predictors, 20 models were constructed without quality control measures reported for predictor assessment, so they were at an unclear risk of bias. In terms of outcomes, 20 models were all at low risk of bias. Regarding statistical analysis, 14 models included in 13 studies [27,29-31,33,35,37,40-43,45,46] were constructed with poor sample size design, missing data handled inappropriately or predictors screened irrationally, so they were at high risk of bias, as shown in Figure 2. The risk of bias in including studies is presented in Table S4 and Table S5 in Multimedia Appendix 1.

**Figure 3:**
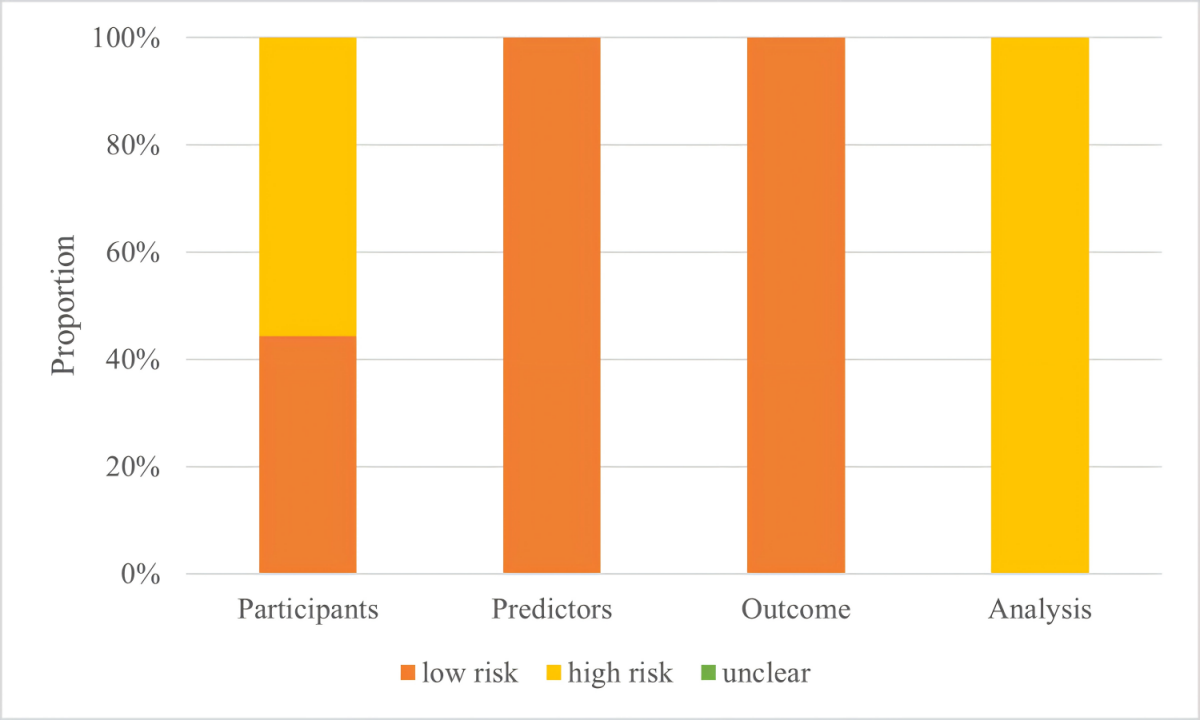
Risk assessment of machine learning models to predict coronary artery lesions in people with Kawasaki disease.

In the early prediction of CALs in people with KD, 5 models [48-52] were based on data from retrospective case-control studies, and therefore had a high risk of participant selection bias. In predictors and outcomes as domains, 9 models were all at low risk of bias. In statistical analysis as a domain, 6 models [47,51-55] were at high risk of bias because of irrational sample size design, and 3 models [48-50] were at high risk of bias owing to incorrect use of internal validation methods as shown in Figure 3.

### Meta-Analysis

#### Differentiation of Kawasaki Disease From Other Febrile Illnesses

**Figure 4:**
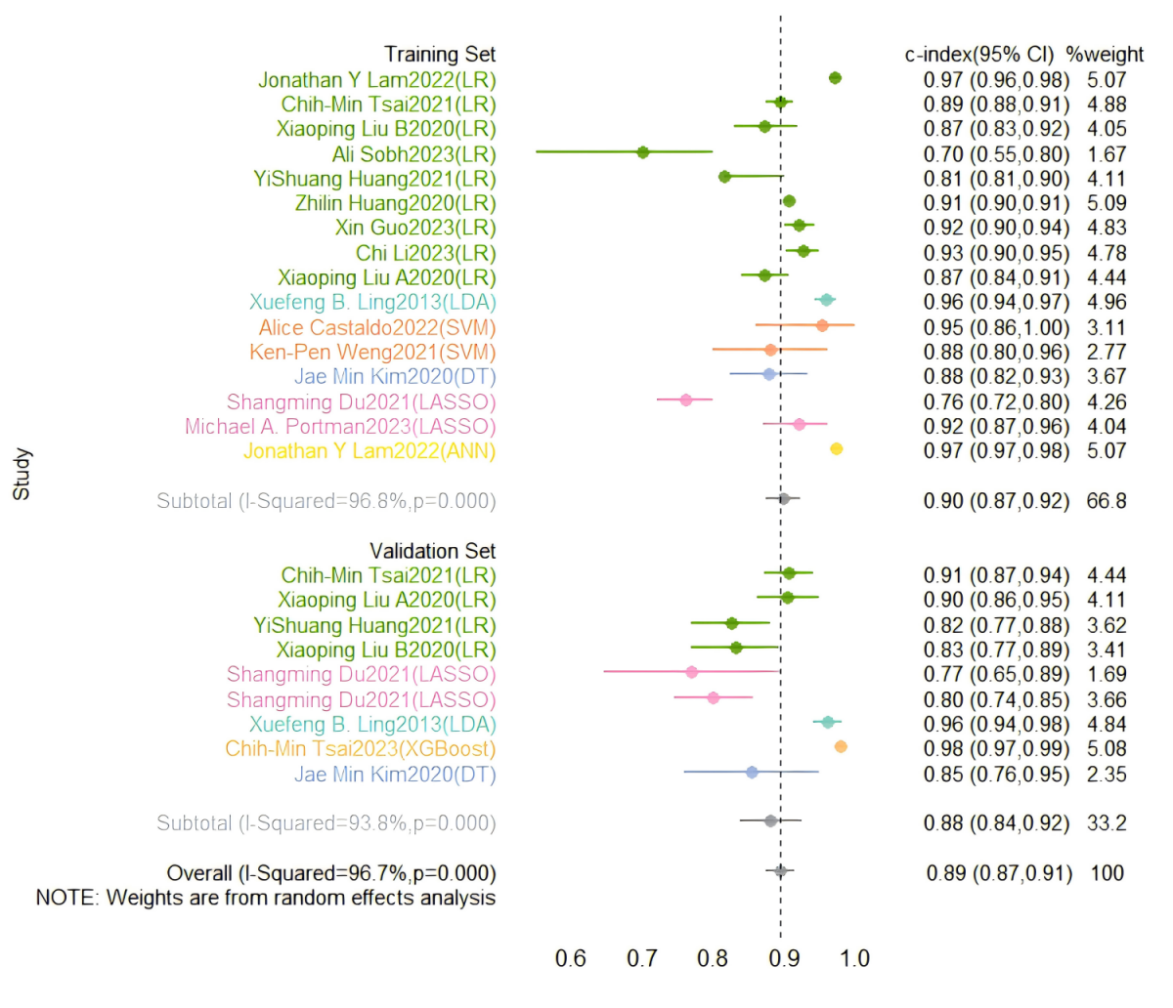
Concordance index forest plots of machine learning to identify Kawasaki disease and febrile controls in training and validation sets [29-36,40-46].

**Figure 5:**
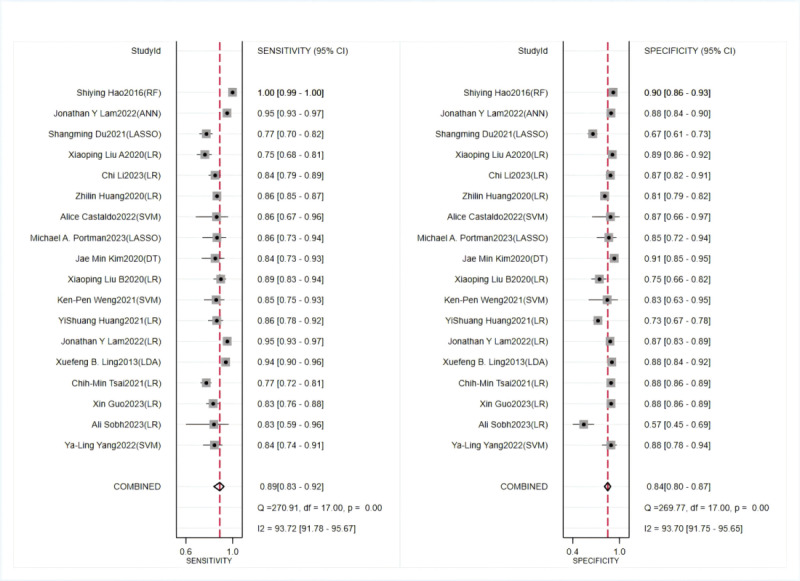
Sensitivity and specificity of machine learning to identify Kawasaki disease and febrile controls in training sets [27,29,30-36,38,40-46].

In the training set, a random-effects model was leveraged for meta-analysis of the C-index, which was 0.898 (95% CI 0.874-0.922), as shown in Figure 4 [29-36,40-46]. The funnel plot showed no significant publication bias among the included studies. The results of meta-analysis for the 2 × 2 table showed a sensitivity of 0.89 (95% CI 0.83-0.92) and a specificity of 0.84 (95% CI 0.80-0.87) in the training set, as shown in Figure 5 [27,29-36,38,40-46].

**Figure 6:**
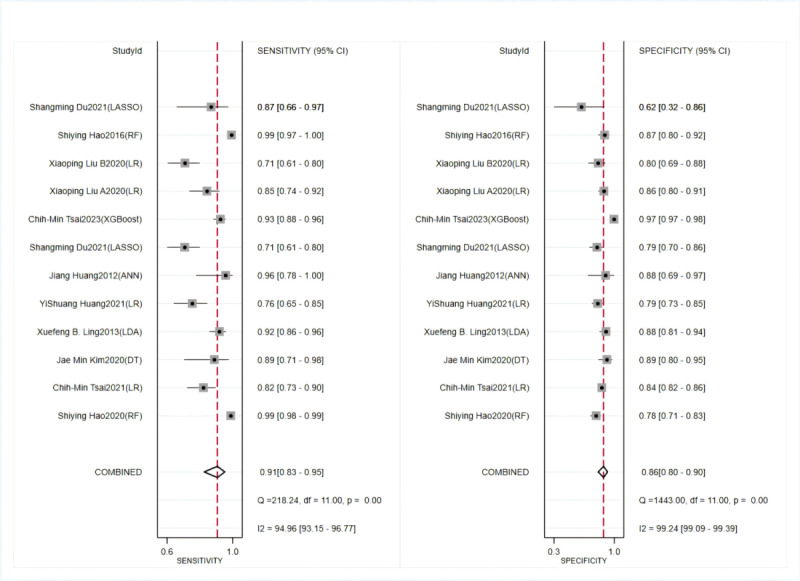
Sensitivity and specificity of machine learning to identify Kawasaki disease and febrile controls in validation sets [28,32-35,37-39,42,44,46].

In the validation set, a random-effects model was used for meta-analysis of the C-index, which was 0.881 (95% CI 0.837-0.925), as shown in Figure 4 [29-36,40-46]. The funnel plot showed that there appeared to be publication bias among the included studies. The results of meta-analysis for the 2 × 2 table showed a sensitivity of 0.91 (95% CI 0.83-0.95) and a specificity of 0.86 (95% CI 0.80-0.90) in the validation set, as shown in Figure 6 [28,32-35,37-39,42,44,46].

#### Prediction of Coronary Artery Lesions in Kawasaki Disease

**Figure 7:**
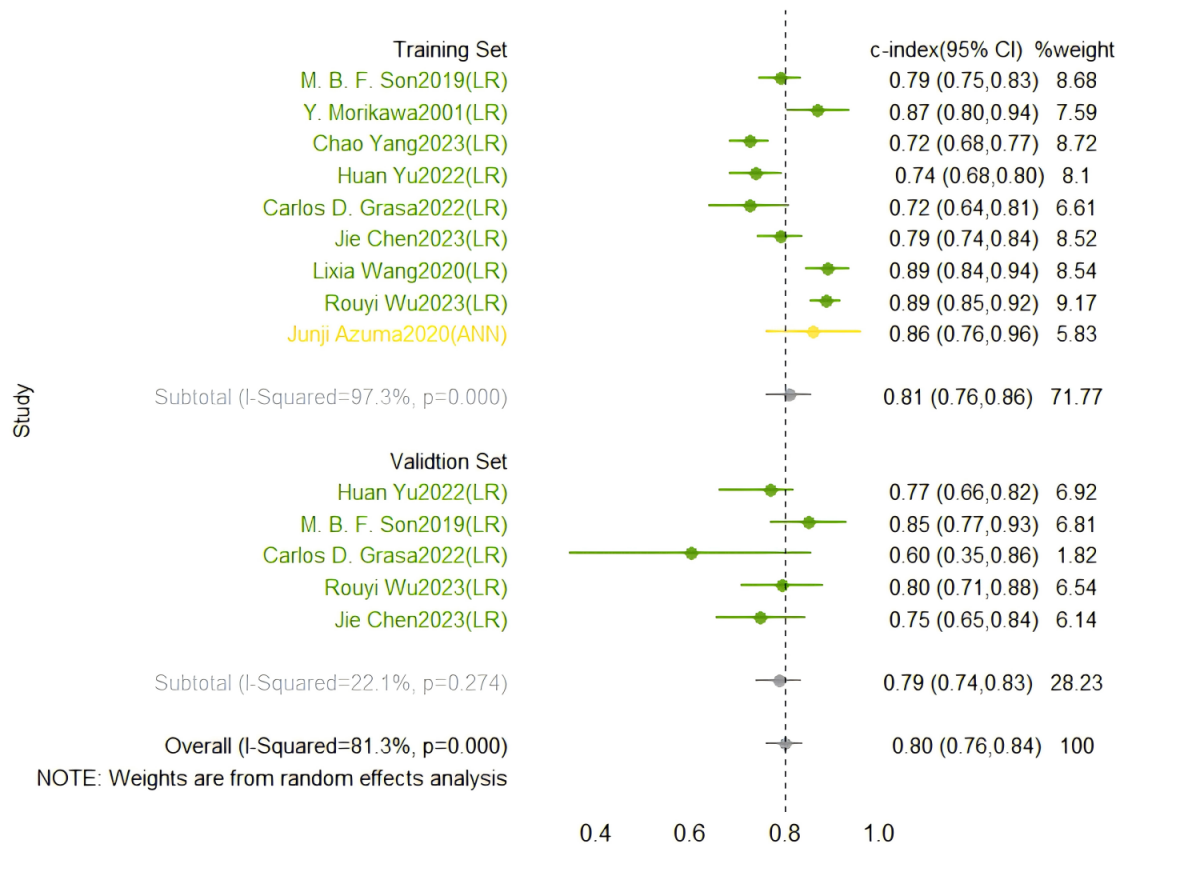
Concordance index forest plots of machine learning to predict coronary artery lesions in Kawasaki disease in training and validation sets [47-55].

**Figure 8:**
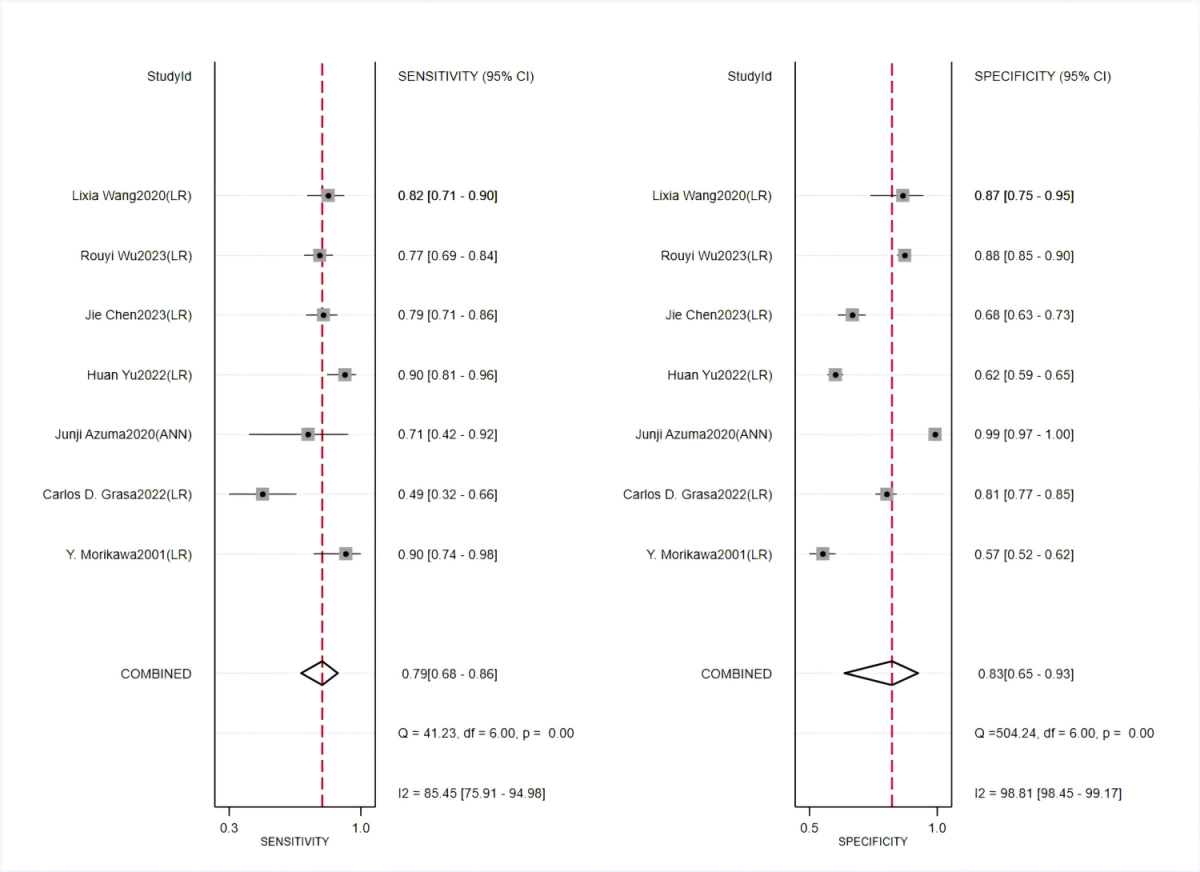
Sensitivity and specificity of machine learning to predict coronary artery lesions in Kawasaki disease in training sets [48-54].

In the training set, a random-effects model was used for meta-analysis of the C-index, which was 0.809 (95% CI 0.761-0.857), as shown in Figure 7 [47-55]. The funnel plot showed no significant publication bias among the included studies. The results of meta-analysis for the 2 × 2 table showed a sensitivity of 0.79 (95% CI 0.68-0.86) and a specificity of 0.83 (95% CI 0.65-0.93) in the training set, as shown in Figure 8 [48-54].

In the validation set, a random-effects model was used for meta-analysis of the C-index, which was 0.787 (95% CI 0.738-0.835), as shown in Figure 7 [47-55]. The funnel plot showed that there appeared to be publication bias among the included studies. Since the number of models in the validation set was less than 4 and did not reach the lower limit for meta-analysis, we only described the ranges of sensitivity and specificity in the validation set, which were 0.220-0.952 and 0.644-0.755, respectively.

### Predictors

#### Frequencies of Use of Diagnostic Factors to Construct ML Models for Differentiation of KD From Other Febrile Illnesses

**Figure 9:**
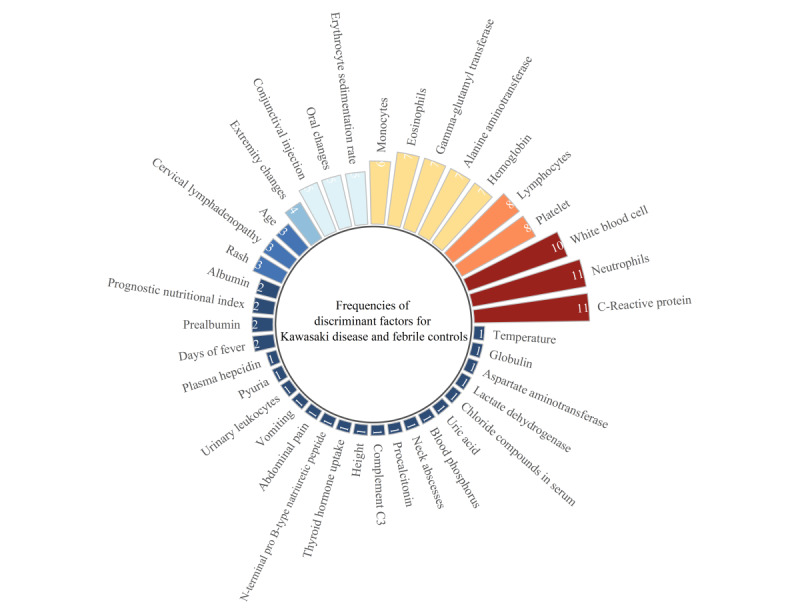
Frequencies of use of diagnostic factors to construct machine learning models to identify Kawasaki disease from febrile controls.

Regarding the use of diagnostic factors for differentiation of KD, there were a total of 39 diagnostic factors including CRP (n=11), neutrophils (NEUT; n=11), WBC (n=10), PLT (n=8), lymphocytes (n=8), hemoglobin (n=7), alanine aminotransferase (n=7), gamma-glutamyl transferase (n=7), eosinophils (n=7), monocytes (n=6), ESR (n=5), oral changes (n=5), conjunctival injection (n=5), extremity changes (n=4), age (n=3), cervical lymphadenopathy (n=3), rash (n=3), albumin (n=2), prognostic nutritional index (n=2), prealbumin (n=2), and duration (days) of fever (n=2). The following diagnostic factors were each used with a frequency of 1: Plasma Hepcidin, Pyuria, Urinary Leukocytes, Vomiting, Abdominal Pain, N-terminal Pro B-type Natriuretic Peptide, Thyroid Hormone Uptake, Height, complement C3, Procalcitonin, Neck Abscesses, Blood Phosphorus, Uric Acid, Chloride Compounds in serum, lactate dehydrogenase, aspartate aminotransferase, globulin, and temperature (Figure 9).

#### Frequencies of Use of Predictors to Construct ML Models for Prediction of Coronary Artery Lesions in KD

**Figure 10:**
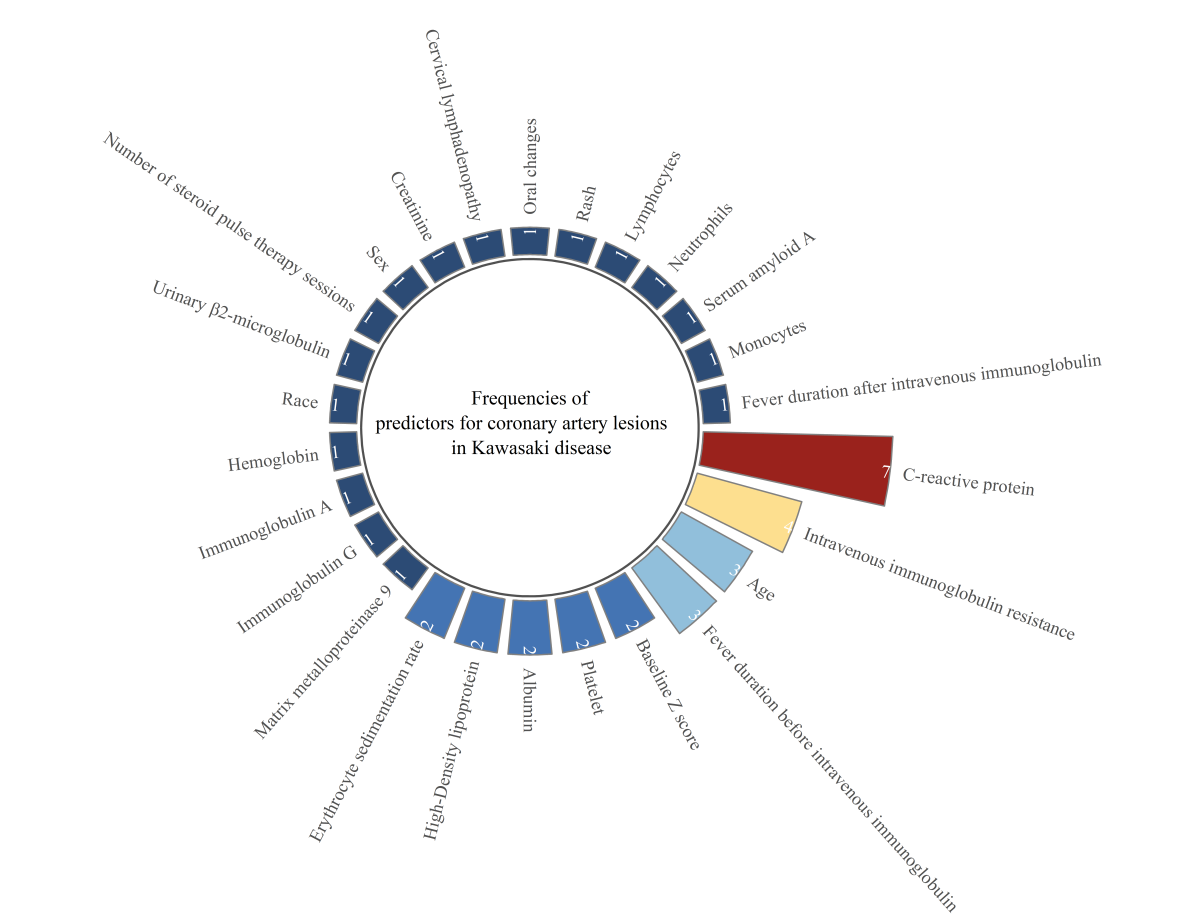
Frequencies of use of predictors to construct machine learning models to predict coronary artery lesions in Kawasaki disease.

Regarding the use of diagnostic factors for prediction of CALs, there were a total of 26 predictors including CRP (n=7), IVIG resistance (n=4), duration of fever before IVIG infusion (n=3), age (n=3), baseline Z score (n=2), high-density Lipoprotein (n=2), albumin (n=2), PLT (n=2), ESR (n=2), duration of fever after IVIG infusion (n=1), monocytes (n=1), serum amyloid A (n=1), NEUT (n=1), lymphocytes (n=1), rash (n=1), oral changes (n=1), cervical lymphadenopathy (n=1), sex (n=1), race (n=1), creatinine (n=1), number of Steroid Pulse therapy sessions (n=1), Urinary β2-microglobulin (n=1), Hgb (n=1), Immunoglobulin A (n=1), immunoglobulin G (n=1), and matrix metalloproteinase 9 (n=1; Figure 10).

## Discussion

### Principal Findings

Our study included a total of 29 ML prediction models, of which 20 models focused on differentiating KD from other febrile illnesses, and 9 models focused on predicting the risk of CALs in children with KD. This study unraveled that ML achieved a favorable performance in differentiating KD from other febrile illnesses and predicting the risk of CALs in children with KD. In the validation set for ML to differentiate KD from other febrile illnesses, the C-index, sensitivity, and specificity were 0.881 (95% CI 0.837-0.925), 0.91 (95% CI 0.83-0.95), and 0.86 (95% CI 0.80-0.90), respectively. In the validation set for early prediction of the risk of CALs in children with KD, the C-index was 0.787 (95% CI 0.738-0.835), with a sensitivity range of 0.220-0.952 and a specificity range of 0.644-0.755. All the results in the validation set versus the training set showed no overfitting.

CRP, NEUT, and WBC were the most frequently used diagnostic factors in the diagnostic model for KD, and CRP was also the most frequent predictor in the predictive model for CALs in people with KD. CRP is a very common laboratory test item. All the models we included were constructed based on interpretable ML models and routine clinical or laboratory variables, suggesting that desirable predictive results can be obtained if common clinical characteristics and laboratory variables are combined with appropriate ML modeling methods.

### Comparison With Previous Reviews

In recent years, ML has been extensively applied in the diagnosis and early prediction of diseases to enhance precision and efficiency in health care, but the value of its application in the diagnosis of KD and early prediction of the risk of CALs in KD children is still unknown. A systematic review of existing studies is necessary to guide the development and application of ML in this field. To our knowledge, our study is the first systematic review evaluating the accuracy of ML for differentiation of KD from other febrile illnesses and for early prediction of CALs in children with KD.

In clinical practice, there is an emphasis on using rapid and cost-effective diagnostic methods for children with fever and at risk of coronary artery disease. Most of the studies included in our review are based on common and routine clinical data. This indicated that when faced with patients with fever, clinicians can use ML methods and the most routine tests to differentiate and diagnose KD.

Over the past few years, many teams have attempted to diagnose KD using genomic technology in addition to common clinical characteristics and laboratory variables. A meta-analysis by Zheng et al [56] showed that total mixed miRNAs detection for diagnosing KD had a sensitivity of 0.7 (0.66-0.74), a specificity of 0.87 (0.83-0.90), and an Summary Receiver Operating Characteristic of 0.8302. In contrast, our analysis showed that ML had a higher diagnostic accuracy. Another meta-analysis [57] showed that compared with ML, ncRNA had a similar diagnostic accuracy with an AUC of 0.90 (0.87-0.92) in differentiating KD from febrile illnesses. Some studies have found the same genetic association with risk for CAL in KD children. Xie et al [58] reviewed 164 previous studies and found that *BTNL2*, *CASP3*, *FCGR2A*, *FGF23*, *FGβ*, *GRIN3A*, *HLA-E*, *IL10*, *ITPKC*, and *TGFBR2* were linked to a high risk for CALs in children with KD. Another meta-analysis [59] showed that Allele A at the functional SNP rs72689236 of the gene encoding caspase-3 was a genetic marker for susceptibility to KD with CALs. Although genomic technology has demonstrated some value and significance in the diagnosis of KD and the prediction of KD with CALs, genetic testing is time consuming and not applicable to the diagnosis of the acute phase of the disease. Considering the higher cost of genomic technology, the need for high-quality control in the laboratory and the complexity of genomic data analysis and interpretation, genomic technology is less clinically applicable to the early diagnosis of KD and the prediction of concomitant CALs. In contrast, common clinical variables are more applicable and convenient in most current clinical settings, while being more conducive to reducing the economic burden on people with KD.

During the application of ML in the field of medicine, model type selection is critical to balance the accuracy and interpretability of a model. Interpretable models, such as logistic regression and the decision tree, tend to have a worse accuracy in identifying outcome measures. However, less interpretable models, such as Random Forest, XGboost, and Artificial Neural Networks, tend to have a higher accuracy. Logistic regression was the main ML model included in our study, suggesting that early diagnosis of KD and early prediction of CALs in people with KD were feasible based on routinely interpretable models.

In addition, the selection of model variables is crucial for the construction of ML models, because it determines the testing accuracy of ML models. In current clinical practice, model variables can be classified into several types below, including common interpretable clinical characteristics, image-based radiomic features, and pathologic characteristics. Considering that it is difficult to avoid excessive imaging equipment configuration and bias in image segmentation during obtainment of variables through imaging and that pathologic characteristics have limitations including difficulty in quantification and limited data interpretation, we should prefer to use common clinical characteristics as model variables among these variables. The ML model variables included in our study were dominated by common interpretable clinical characteristics, which suggests that easy-to-use clinical tools can be developed in the future based on common clinical characteristics.

### Advantages and Limitations of the Study

Our study is the first systematic review evaluating the accuracy of ML for the differentiation of KD from other febrile illnesses and for early prediction of CALs in children with KD. It was aimed at providing medical evidence for this field. However, our study has the following limitations. First, despite an extensive search, the number of original studies included is still limited, thereby resulting in a failure to conduct effective subgroup analysis based on ML model types in our study. Second, most of the ML models included in our review were constructed using random sampling for internal validation, with only a few studies using an independent external validation set to verify the accuracy of the models. This limitation somewhat restricts the generalizability and interpretation of our results. Third, we did not quantitatively describe the associations between the predictors (model variables) and KD or CALs in KD.

### Conclusions

ML is desirably effective in differentiating KD from other febrile illnesses, and it is also able to fairly predict the occurrence of CALs. However, the development of ML models in the related field is still limited by a small amount of training data, its low quality and a single validation method. Thus, more subsequent multicenter studies are needed to provide more diverse data for ML models, enhance data quality, improve the validation method and outcome measures for ML models, refine the existing models, and develop new high-quality models. In future work, a quantitative characterization of the association between predictors (model variables) and both KD and CALs in KD is needed. In the meantime, more multicenter studies are desired to validate our conclusions. Furthermore, we plan to explore how to further validate and apply ML technologies in clinical practice.

## Appendix

Multimedia Appendix 1 Additional tables.

Multimedia Appendix 2 PRISMA checklist.

## Abbreviations

AI: artificial intelligence
C-index: concordance index
CAL: coronary artery lesion
CRP: C-reactive protein
ESR: erythrocyte sedimentation rate
IVIG: intravenous immunoglobulin
KD: Kawasaki disease
ML: machine learning
NEUT: neutrophils
PLT: platelet
PROBAST: the Prediction Model Risk of Bias Assessment Tool
WBC: white blood cell
