# Supplementary material for: Accuracy of Machine Learning in Discriminating Kawasaki Disease and Other Febrile Illnesses: Systematic Review and Meta-Analysis
Source: J Med Internet Res. 2024 Nov 18;26:e57641. doi: 10.2196/57641 (PMC11612596; doi:10.2196/57641)
Supplement: Multimedia Appendix 1 [file jmir_v26i1e57641_app1.docx]

**Table S1:** Literature search strategy.

**1.Pubmed**

| Search number | Query | Results |
| --- | --- | --- |
| #1 | "Mucocutaneous Lymph Node Syndrome"[Mesh] | 7,349 |
| #2 | ((((((((Mucocutaneous Lymph Node Syndrome[Title/Abstract]) OR (Kawasaki Syndrome[Title/Abstract])) OR (Kawasaki Disease[Title/Abstract])) OR (Kawasaki's disease[Title/Abstract])) OR (Kawasaki's syndrome[Title/Abstract])) OR (MCLS[Title/Abstract])) OR (muco-cutaneous lymph node syndrome[Title/Abstract])) OR (Kawasaki disease shock syndrome[Title/Abstract])) OR (mucocutaneous lymphnode syndrome[Title/Abstract]) | 9,723 |
| #3 | ("Mucocutaneous Lymph Node Syndrome"[Mesh]) OR (((((((((Mucocutaneous Lymph Node Syndrome[Title/Abstract]) OR (Kawasaki Syndrome[Title/Abstract])) OR (Kawasaki Disease[Title/Abstract])) OR (Kawasaki's disease[Title/Abstract])) OR (Kawasaki's syndrome[Title/Abstract])) OR (MCLS[Title/Abstract])) OR (muco-cutaneous lymph node syndrome[Title/Abstract])) OR (Kawasaki disease shock syndrome[Title/Abstract])) OR (mucocutaneous lymphnode syndrome[Title/Abstract])) | 10,260 |
| #4 | "Machine Learning"[Mesh] | 60,193 |
| #5 | (((((((((((((((((((((((((machine learning[Title/Abstract]) OR (Transfer Learning[Title/Abstract])) OR (Deep learning[Title/Abstract])) OR (Ensemble Learning[Title/Abstract])) OR (artificial intelligence[Title/Abstract])) OR (random forest[Title/Abstract])) OR (neural network[Title/Abstract])) OR (neural networks[Title/Abstract])) OR (K-Nearest Neighbor[Title/Abstract])) OR (CNN[Title/Abstract])) OR (Support vector machine[Title/Abstract])) OR (SVM[Title/Abstract])) OR (Gradient Boosting Machine[Title/Abstract])) OR (Nomogram[Title/Abstract])) OR (XGBoost[Title/Abstract])) OR (Adaboost[Title/Abstract])) OR (Decision tree[Title/Abstract])) OR (ResNet-50[Title/Abstract])) OR (ResNet[Title/Abstract])) OR (Naive Bayesian[Title/Abstract])) OR (Multilayer perceptron[Title/Abstract])) OR (Bayesian network[Title/Abstract])) OR (Radiomics[Title/Abstract])) OR (Radiomic[Title/Abstract])) OR (Prediction model[Title/Abstract])) OR (Risk model[Title/Abstract]) | 304,144 |
| #6 | ("Machine Learning"[Mesh]) OR ((((((((((((((((((((((((((machine learning[Title/Abstract]) OR (Transfer Learning[Title/Abstract])) OR (Deep learning[Title/Abstract])) OR (Ensemble Learning[Title/Abstract])) OR (artificial intelligence[Title/Abstract])) OR (random forest[Title/Abstract])) OR (neural network[Title/Abstract])) OR (neural networks[Title/Abstract])) OR (K-Nearest Neighbor[Title/Abstract])) OR (CNN[Title/Abstract])) OR (Support vector machine[Title/Abstract])) OR (SVM[Title/Abstract])) OR (Gradient Boosting Machine[Title/Abstract])) OR (Nomogram[Title/Abstract])) OR (XGBoost[Title/Abstract])) OR (Adaboost[Title/Abstract])) OR (Decision tree[Title/Abstract])) OR (ResNet-50[Title/Abstract])) OR (ResNet[Title/Abstract])) OR (Naive Bayesian[Title/Abstract])) OR (Multilayer perceptron[Title/Abstract])) OR (Bayesian network[Title/Abstract])) OR (Radiomics[Title/Abstract])) OR (Radiomic[Title/Abstract])) OR (Prediction model[Title/Abstract])) OR (Risk model[Title/Abstract])) | 309,244 |
| #7 | #2 AND #4 AND #6 | 88 |

**2.Cochrane**

| Search number | Query | Results |
| --- | --- | --- |
| #1 | MeSH descriptor: [Mucocutaneous Lymph Node Syndrome] explode all trees | 145 |
| #2 | (Mucocutaneous Lymph Node Syndrome):ti,ab,kw OR (Kawasaki Syndrome):ti,ab,kw OR (Kawasaki Disease):ti,ab,kw OR (Kawasaki's disease):ti,ab,kw OR (Kawasaki's syndrome):ti,ab,kw | 350 |
| #3 | (MCLS):ti,ab,kw OR (muco-cutaneous lymph node syndrome):ti,ab,kw OR (Kawasaki disease shock syndrome):ti,ab,kw OR (mucocutaneous lymphnode syndrome):ti,ab,kw | 24 |
| #4 | #1 or #2 or #3 | 366 |
| #5 | MeSH descriptor: [Machine Learning] explode all trees | 920 |
| #6 | (Transfer Learning):ti,ab,kw OR (Deep learning):ti,ab,kw OR (Ensemble Learning):ti,ab,kw OR (artificial intelligence):ti,ab,kw OR (random forest):ti,ab,kw | 5090 |
| #7 | (neural network):ti,ab,kw OR (neural networks):ti,ab,kw OR (K-Nearest Neighbor):ti,ab,kw OR (CNN):ti,ab,kw OR (Support vector machine):ti,ab,kw | 3828 |
| #8 | (SVM):ti,ab,kw OR (Gradient Boosting Machine):ti,ab,kw OR (Nomogram):ti,ab,kw OR (XGBoost):ti,ab,kw OR (Adaboost):ti,ab,kw | 1983 |
| #9 | (Decision tree):ti,ab,kw OR (ResNet-50):ti,ab,kw OR (ResNet):ti,ab,kw OR (Naive Bayesian):ti,ab,kw OR (Multilayer perceptron):ti,ab,kw | 1088 |
| #10 | (Bayesian network):ti,ab,kw OR (Radiomics):ti,ab,kw OR (Radiomic):ti,ab,kw OR (Prediction model):ti,ab,kw OR (Risk model):ti,ab,kw | 33712 |
| #11 | (machine learning):ti,ab,kw | 2613 |
| #12 | #5 or #6 or #7 or #8 or #9 or #10 or #11 | 42904 |
| #13 | #4 and #12 | 8 |
| #14 |  |  |
| #15 |  |  |
| #16 |  |  |
| #17 |  |  |
| #18 |  |  |
| #19 |  |  |
| #20 |  |  |
| #21 |  |  |
| #22 |  |  |

**3.Embase**

| Search number | Query | Results |
| --- | --- | --- |
| #1 | 'mucocutaneous lymph node syndrome'/exp | 14694 |
| #2 | 'mucocutaneous lymph node syndrome':ab,ti OR 'kawasaki syndrome':ab,ti OR 'kawasaki disease':ab,ti OR 'kawasakis disease':ab,ti OR 'kawasakis syndrome':ab,ti OR mcls:ab,ti OR 'muco-cutaneous lymph node syndrome':ab,ti OR 'kawasaki disease shock syndrome':ab,ti OR 'mucocutaneous lymphnode syndrome':ab,ti | 12865 |
| #3 | #1 OR #2 | 16306 |
| #4 | 'machine learning'/exp | 417430 |
| #5 | 'machine learning':ab,ti OR 'transfer learning':ab,ti OR 'deep learning':ab,ti OR 'ensemble learning':ab,ti OR 'artificial intelligence':ab,ti OR 'random forest':ab,ti OR 'neural network':ab,ti OR 'neural networks':ab,ti OR 'k-nearest neighbor':ab,ti OR cnn:ab,ti OR 'support vector machine':ab,ti OR svm:ab,ti OR 'gradient boosting machine':ab,ti OR nomogram:ab,ti OR xgboost:ab,ti OR adaboost:ab,ti OR 'decision tree':ab,ti OR 'resnet 50':ab,ti OR resnet:ab,ti OR 'naive bayesian':ab,ti OR 'multilayer perceptron':ab,ti OR 'bayesian network':ab,ti OR radiomics:ab,ti OR radiomic:ab,ti OR 'prediction model':ab,ti OR 'risk model':ab,ti | 353568 |
| #6 | #4 OR #5 | 565957 |
| #7 | #3 AND #6 | 150 |

**4.Web of science**

| Search number | Query | Results |
| --- | --- | --- |
| #1 | Mucocutaneous Lymph Node Syndrome (Topic) OR Kawasaki Syndrome (Topic) OR Kawasaki Disease (Topic) OR Kawasaki's disease (Topic) OR Kawasaki's syndrome (Topic) OR MCLS (Topic) OR muco-cutaneous lymph node syndrome (Topic) OR Kawasaki disease shock syndrome (Topic) OR mucocutaneous lymphnode syndrome (Topic) and Preprint Citation Index (Exclude – Database) | 16483 |
| #2 | machine learning (Topic) OR Transfer Learning (Topic) OR Deep learning (Topic) OR Ensemble Learning (Topic) OR artificial intelligence (Topic) OR random forest (Topic) OR neural network (Topic) OR neural networks (Topic) OR K-Nearest Neighbor (Topic) OR CNN (Topic) OR Support vector machine (Topic) OR SVM (Topic) OR Gradient Boosting Machine (Topic) OR Nomogram (Topic) OR XGBoost (Topic) OR Adaboost (Topic) OR Decision tree (Topic) OR ResNet-50 (Topic) OR ResNet (Topic) OR Naive Bayesian (Topic) OR Multilayer perceptron (Topic) OR Bayesian network (Topic) OR Radiomics (Topic) OR Radiomic (Topic) OR Prediction model (Topic) OR Risk model (Topic) and Preprint Citation Index (Exclude – Database) | 3931065 |
| #3 | #1 AND #2 and Preprint Citation Index (Exclude – Database) | 569 |
| #4 |  |  |
| #5 |  |  |
| #6 |  |  |

**Table S2:** Characteristics of Machine Learning Studies to Identify Kawasaki Disease from Other Febrile Diseases.

| No. | First author | Year of publication | Author's nationality | Study type, data source | Number of KD patients in the study | Total number of patients in the study | Validation set generation method | Methods for variable screening | Methods for handling missing values | Type of a model used |
| --- | --- | --- | --- | --- | --- | --- | --- | --- | --- | --- |
| 1 | Shiying Hao [38] | 2016 | USA | Prospective, multicenter | 801 | 1280 | Random sampling, external validation | Univariate analysis+Multivariate Analysis | Weighted K-nearest neighbors | Linear Discriminant Analysis |
| 1 | Shiying Hao [39] | 2020 | USA | Prospective, single-center | 982 | 1192 | External validation by Shiying Hao in 2016 | Univariate analysis+Multivariate Analysis | No missing values | Linear Discriminant Analysis |
| 2 | Ya-Ling Yang [27] | 2022 | China | Retrospective, single-center | 115 | 219 | Random sampling (7:3), five-fold cross-validation (CV) | Univariate analysis+Multivariate Analysis | No missing values | Support Vector Machine |
| 3 | Chih-Min Tsai [28] | 2023 | China | Retrospective, multicenter | 1243 | 74742 | Internal validation (random sampling, 8:2) + external validation (multicenter) | Univariate analysis+Multivariate Analysis | No missing values | XGBoost |
| 4 | Ali Sobh [43] | 2023 | Egypt | Prospective, single-center | 18 | 90 | NA | Univariate analysis+Multivariate Analysis | No missing values | Logistic Regression |
| 5 | Michael A. Portman [40] | 2023 | USA | Unknown, single-center | 50 | 150 | 10-fold cross-validation | Univariate analysis+Multivariate Analysis | No missing values | Least Absolute Shrinkage and Selection Operator |
| 6 | Chi Li [29] | 2023 | China | Retrospective, single-center | 299 | 608 | Random sampling (7:3) | Univariate analysis+Multivariate Analysis | No missing values | Logistic Regression |
| 7 | Xin Guo [30] | 2023 | China | Retrospective, single-center | 190 | 1794 | NA | Univariate analysis+Multivariate Analysis | No missing values | Logistic Regression |
| 8 | Jonathan Y Lam [41] (including two models) | 2022 | USA | Prospective, multicenter | 775 | 1448 | Random sampling (8:2), 10-fold cross validation | Univariate analysis+Multivariate Analysis | Exclude records with missing values | Artificial Neural Network, Logistic Regression |
| 9 | [Shangming Du](https://www.frontiersin.org/people/u/1452433) [44] | 2021 | Germany | Prospective, registered database | 323 | 744 | Random sampling (7:3), 10-fold cross validation, external validation | Univariate analysis+Multivariate Analysis | No missing values | Least Absolute Shrinkage and Selection Operator |
| 10 | Alice Castaldo [45] | 2022 | Italy | Unknown, single-center | 28 | 144 | Cross validation run 100 times, external validation | Univariate analysis+Multivariate Analysis | No missing values | Support Vector Machine |
| 11 | Ken-Pen Weng [31] | 2021 | China | Unknown, single-center | 68 | 92 | Cross validation run 10 times | Univariate analysis+Multivariate Analysis | Exclude records with missing values | Support Vector Machine |
| 12 | Chih-Min Tsai [32] | 2021 | China | Retrospective, single-center | 758 | 7068 | 5-fold cross validation, external validation: multicenter | Univariate analysis+Multivariate Analysis | No missing values | Logistic Regression |
| 13 | Yishuang Huang [33] | 2021 | China | Retrospective, single-center | 198 | 692 | Random sampling (6:4) | Univariate analysis+Multivariate Analysis | Exclude records with missing values | Logistic Regression |
| 14 | Xiaoping Liu A [34] | 2020 | China | Prospective, single-center | 249 | 800 | Random sampling (7:3), external validation: multicenter | Univariate analysis+Multivariate Analysis | No missing values | Logistic Regression |
| 15 | Xiaoping Liu B [35] | 2020 | China | Retrospective, single-center | 227 | 420 | Random sampling | Univariate analysis+Multivariate Analysis | No missing values | Logistic Regression |
| 16 | Jae Min Kim [46] | 2020 | Republic of Korea | Retrospective, single-center | 85 | 309 | Random sampling (2:1) | Univariate analysis | No missing values | Decision Tree |
| 17 | Zhilin Huang [36] | 2020 | China | Retrospective, single-center | 5642 | 11176 | 5-fold cross validation | Univariate analysis+Multivariate Analysis | Multiple Imputation | Logistic Regression |
| 18 | Jiang Huang [37] | 2012 | China | Unknown, multicenter | 78 | 138 | Random sampling | Univariate analysis+Multivariate Analysis | No missing values | Artificial Neural Network |
| 19 | Xuefeng B. Ling [42] | 2013 | USA | Prospective, single-center | 412 | 776 | Random sampling | Univariate analysis+Multivariate Analysis | No missing values | Linear Discriminant Analysis |

**Table S3:** Characteristics of Machine Learning Studies to Predict Coronary Artery Lesions in People with Kawasaki Disease.

| No. | First author | Year of publication | Author's nationality | Study type | Data source | Number of patients with CALs | Total number of KD patients | Validation set generation method | Methods for variable screening | Methods for handling missing values | Type of a model used |
| --- | --- | --- | --- | --- | --- | --- | --- | --- | --- | --- | --- |
| 1 | Chao Yang [47] | 2023 | China | Prospective | Single-center | 218 | 568 | Bootstrap (1000 repetitions of sampling) | Univariate analysis+Multivariate Analysis | Impute missing values using the mean | Logistic Regression |
| 2 | Rouyi Wu [48] | 2023 | China | Retrospective | Single-center | 176 | 952 | Random sampling | Univariate analysis+Multivariate Analysis | Impute missing values using the mean | Logistic Regression |
| 3 | Jie Chen [49] | 2023 | China | Retrospective | Single-center | 147 | 599 | Random sampling (3:1) | Univariate analysis+Multivariate Analysis | No missing values | Logistic Regression |
| 4 | Huan Yu [50] | 2022 | China | Retrospective | Multicenter | 119 | 1749 | Random sampling (2:1) | Univariate analysis+Multivariate Analysis | No missing values | Logistic Regression |
| 5 | Carlos D. Grasa [54] | 2022 | Spain | Prospective | Multicenter | 63 | 614 | External validation: prospective, multicenter | Multivariate Analysis | Multiple Imputation | Logistic Regression |
| 6 | [Lixia Wang](https://journals.sagepub.com/doi/abs/10.1136/jim-2020-001281#con1) [51] | 2020 | China | Retrospective | Single-center | 76 | 130 | Internal validation | Univariate analysis+Multivariate Analysis | No missing values | Logistic Regression |
| 7 | [Junji Azuma](https://www.nature.com/articles/s41598-020-68657-0#auth-Junji-Azuma-Aff1) [52] | 2020 | Japan | Retrospective | Single-center | 14 | 352 | 5-fold cross validation, external validation | Univariate analysis+Multivariate Analysis | No missing values | Logistic Regression |
| 8 | M. B. F. Son [55] | 2019 | USA | Prospective | Multicenter | 142 | 1088 | Random sampling | Univariate analysis+Multivariate Analysis | Exclude records with missing values | Logistic Regression |
| 9 | Y. Morikawa [53] | 2001 | Japan | Prospective | Multicenter | 31 | 451 | Random sampling, subsampling repeated 100 times | Univariate analysis+Multivariate Analysis | Exclude records with missing values | Logistic Regression |

**Table S4:** Risk of bias in Machine Learning Studies to Predict Coronary Artery Lesions in People with Kawasaki Disease.

| No | Author | Participants | | Predictors | | | Outcome | | | | | | Analysis | | | | | | | | |
| --- | --- | --- | --- | --- | --- | --- | --- | --- | --- | --- | --- | --- | --- | --- | --- | --- | --- | --- | --- | --- | --- |
|  |  | Q1 | Q2 | Q1 | Q2 | Q3 | Q1 | Q2 | Q3 | Q4 | Q5 | Q6 | Q1 | Q2 | Q3 | Q4 | Q5 | Q6 | Q7 | Q8 | Q9 |
| 1 | S. Y. Hao [38,39] | Low | Low | Low | Unclear | Low | Low | Low | Low | Low | Low | Low | Low | Low | Low | Low | Low | Low | Low | Low | Low |
| 2 | Y. L. Yang [27] | High | Low | Low | Unclear | Low | Low | Low | Low | Low | Low | Low | High | Low | Low | Low | Low | Low | Low | Low | Low |
| 3 | C. M. Tsai [28] | High | Low | Low | Unclear | Low | Low | Low | Low | Low | Low | Low | Low | Low | Low | Low | Low | Low | Low | Low | Low |
| 4 | A. Sobh [43] | Low | Low | Low | Unclear | Low | Low | Low | Low | Low | Low | Low | High | Low | Low | Low | Low | Low | Low | High | Low |
| 5 | M. A. Portman [40] | Unclear | Low | Low | Unclear | Low | Low | Low | Low | Low | Low | Low | High | Low | Low | Low | Low | Low | Low | Low | Low |
| 6 | C. Li [29] | High | Low | Low | Unclear | Low | Low | Low | Low | Low | Low | Low | High | Low | Low | Low | Low | Low | Low | High | Low |
| 7 | X. Guo [30] | High | Low | Low | Unclear | Low | Low | Low | Low | Low | Low | Low | High | Low | Low | Low | Low | Low | Low | Unclear | Low |
| 8 | J. Y. Lam [41] | Low | Low | Low | Unclear | Low | Low | Low | Low | Low | Low | Low | High | Low | Low | High | Low | Low | Low | Low | Low |
| 8 | J. Y. Lam [41] | Low | Low | Low | Unclear | Low | Low | Low | Low | Low | Low | Low | High | Low | Low | High | Low | Low | Low | Low | Low |
| 9 | S. M. Du [44] | Low | Low | Low | Unclear | Low | Low | Low | Low | Low | Low | Low | Low | Low | Low | Low | Low | Low | Low | Low | Low |
| 10 | A. Castaldo [45] | Unclear | Low | Low | Unclear | Low | Low | Low | Low | Low | Low | Low | High | Low | Low | Low | Low | Low | Low | Low | Low |
| 11 | K. P. Weng [31] | Unclear | Low | Low | Unclear | Low | Low | Low | Low | Low | Low | Low | High | Low | Low | High | Low | Low | Low | Low | Low |
| 12 | C. M. Tsai [32] | High | Low | Low | Unclear | Low | Low | Low | Low | Low | Low | Low | Low | Low | Low | Low | Low | Low | Low | Low | Low |
| 13 | Y. S. Huang [33] | High | Low | Low | Unclear | Low | Low | Low | Low | Low | Low | Low | Low | Low | Low | High | Low | Low | Low | High | Low |
| 14 | X. P. Liu [34] | Low | Low | Low | Unclear | Low | Low | Low | Low | Low | Low | Low | Low | Low | Low | Low | Low | Low | Low | Low | Low |
| 15 | X. P. Liu [35] | High | Low | Low | Unclear | Low | Low | Low | Low | Low | Low | Low | Low | Low | Low | Low | Low | Low | Low | High | Low |
| 16 | J. M. Kim [46] | High | Low | Low | Unclear | Low | Low | Low | Low | Low | Low | Low | Low | Low | Low | Low | High | Low | Low | Low | Low |
| 17 | Z. L. Huang [36] | High | Low | Low | Unclear | Low | Low | Low | Low | Low | Low | Low | Low | Low | Low | Low | Low | Low | Low | Low | Low |
| 18 | J. Huang [37] | Unclear | Low | Low | Unclear | Low | Low | Low | Low | Low | Low | Low | High | Low | Low | Low | Low | Low | Low | High | Low |
| 19 | X. F. B. Ling [42] | Low | Low | Low | Unclear | Low | Low | Low | Low | Low | Low | Low | Low | Low | Low | Low | Low | Low | Low | High | Low |

Note:

(1)In the “Participants” domain, Q1 : “Were appropriate data sources used, e.g., cohort, RCT, or nested case-control study data? ”

(2) In the “Participants” domain, Q2 : “Were all inclusions and exclusions of participants appropriate?” (3) In the “Predictors” domain, Q1 : “Were predictors defined and assessed in a similar way for all participants?”

(4) In the “Predictors” domain, Q2 : “Were predictor assessments made without knowledge of outcome data?”

(5) In the “Predictors” domain, Q3 : “Are all predictors available at the time the mode is intended to be used?”

(6) In the “Outcome” domain, Q1 : “Was the outcome determined appropriately?”

(7) In the “Outcome” domain, Q2 : “Was a prespecified or standard outcome definition used?”

(8) In the “Outcome” domain, Q3 : “Were predictors excluded from the outcome definition?”

(9) In the “Outcome” domain, Q4 : “Was the outcome defined and determined in a similar way for all participants?”

(10) In the “Outcome” domain, Q5 : “Was the outcome determined without knowledge of predictor information?”

(11) In the “Outcome” domain, Q6 : “Was the time Interval between predictor assessment and outcome determination appropriate?”

(12) In the “Analysis” domain, Q1 : “Were there a reasonable number of participants with the outcome?”

(13) In the “Analysis” domain, Q2 : “Were continuous and categorical predictors handled appropriately?”

(14) In the “Analysis” domain, Q3 : “Were all enrolled participants included in the analysis?”

(15) In the “Analysis” domain, Q4 : “Were participants with missing data handled appropriately?”

(16) In the “Analysis” domain, Q5 : “Was selection of predictors based on univariable analysis avoided?”

(17) In the “Analysis” domain, Q6 : “Were complexities in the data (e.g., censoring, competing risks, sampling of control participants) accounted for appropriately?”

(18) In the “Analysis” domain, Q7 : “Were relevant model performance measures evaluated appropriately?”

(19) In the “Analysis” domain, Q8 : “Were model overfitting underfitting, and optimism in model performance accounted for?”

(20) In the “Analysis” domain, Q9: “Do predictors and their assigned weights in the final model correspond to the results from the reported multivariable analysis?”

**Table S5:** Risk of bias in Machine Learning Studies to Predict Coronary Artery Lesions in People with Kawasaki Disease.

| No | Author | Participants | | Predictors | | | Outcome | | | | | | Analysis | | | | | | | | |
| --- | --- | --- | --- | --- | --- | --- | --- | --- | --- | --- | --- | --- | --- | --- | --- | --- | --- | --- | --- | --- | --- |
|  |  | Q1 | Q2 | Q1 | Q2 | Q3 | Q1 | Q2 | Q3 | Q4 | Q5 | Q6 | Q1 | Q2 | Q3 | Q4 | Q5 | Q6 | Q7 | Q8 | Q9 |
| 1 | C. Yang [47] | Low | Low | Low | Low | Low | Low | Low | Low | Low | Low | Low | High | Low | Low | High | Low | Low | Low | Low | Low |
| 2 | R. Wu [48] | High | Low | Low | Low | Low | Low | Low | Low | Low | Low | Low | Low | Low | Low | High | Low | Low | Low | High | Low |
| 3 | J. Chen [49] | High | Low | Low | Low | Low | Low | Low | Low | Low | Low | Low | Low | Low | Low | Low | Low | Low | Low | High | Low |
| 4 | H. Yu [50] | High | Low | Low | Low | Low | Low | Low | Low | Low | Low | Low | Low | Low | Low | Low | Low | Low | Low | High | Low |
| 5 | C. D. Grasa [54] | Low | Low | Low | Low | Low | Low | Low | Low | Low | Low | Low | High | Low | Low | Low | Low | Low | Low | Low | Low |
| 6 | L. X. Wang [51] | High | Low | Low | Low | Low | Low | Low | Low | Low | Low | Low | High | Low | Low | Low | Low | Low | Low | Low | Low |
| 7 | J. Azuma [52] | High | Low | Low | Low | Low | Low | Low | Low | Low | Low | Low | High | Low | Low | Low | Low | Low | Low | Low | Low |
| 8 | M. B. F. Son [55] | Low | Low | Low | Low | Low | Low | Low | Low | Low | Low | Low | High | Low | Low | High | Low | Low | Low | High | Low |
| 9 | Y. Morikawa [53] | Low | Low | Low | Low | Low | Low | Low | Low | Low | Low | Low | High | Low | Low | Low | Low | Low | Low | Low | Low |

Note:

(1)In the “Participants” domain, Q1 : “Were appropriate data sources used, e.g., cohort, RCT, or nested case-control study data? ”

(2) In the “Participants” domain, Q2 : “Were all inclusions and exclusions of participants appropriate?” (3) In the “Predictors” domain, Q1 : “Were predictors defined and assessed in a similar way for all participants?”

(4) In the “Predictors” domain, Q2 : “Were predictor assessments made without knowledge of outcome data?”

(5) In the “Predictors” domain, Q3 : “Are all predictors available at the time the mode is intended to be used?”

(6) In the “Outcome” domain, Q1 : “Was the outcome determined appropriately?”

(7) In the “Outcome” domain, Q2 : “Was a prespecified or standard outcome definition used?”

(8) In the “Outcome” domain, Q3 : “Were predictors excluded from the outcome definition?”

(9) In the “Outcome” domain, Q4 : “Was the outcome defined and determined in a similar way for all participants?”

(10) In the “Outcome” domain, Q5 : “Was the outcome determined without knowledge of predictor information?”

(11) In the “Outcome” domain, Q6 : “Was the time Interval between predictor assessment and outcome determination appropriate?”

(12) In the “Analysis” domain, Q1 : “Were there a reasonable number of participants with the outcome?”

(13) In the “Analysis” domain, Q2 : “Were continuous and categorical predictors handled appropriately?”

(14) In the “Analysis” domain, Q3 : “Were all enrolled participants included in the analysis?”

(15) In the “Analysis” domain, Q4 : “Were participants with missing data handled appropriately?”

(16) In the “Analysis” domain, Q5 : “Was selection of predictors based on univariable analysis avoided?”

(17) In the “Analysis” domain, Q6 : “Were complexities in the data (e.g., censoring, competing risks, sampling of control participants) accounted for appropriately?”

(18) In the “Analysis” domain, Q7 : “Were relevant model performance measures evaluated appropriately?”

(19) In the “Analysis” domain, Q8 : “Were model overfitting underfitting, and optimism in model performance accounted for?”

(20) In the “Analysis” domain, Q9 : “Do predictors and their assigned weights in the final model correspond to the results from the reported multivariable analysis?”
